# MOV10 binding circ-DICER1 regulates the angiogenesis of glioma via miR-103a-3p/miR-382-5p mediated ZIC4 expression change

**DOI:** 10.1186/s13046-018-0990-1

**Published:** 2019-01-08

**Authors:** Qianru He, Lini Zhao, Xiaobai Liu, Jian Zheng, Yunhui Liu, Libo Liu, Jun Ma, Heng Cai, Zhen Li, Yixue Xue

**Affiliations:** 1 Department of Neurobiology, School of Life Sciences, China Medical University, Shenyang, 110122 People’s Republic of China; 2 Key Laboratory of Cell Biology, Ministry of Public Health of China, China Medical University, Shenyang, 110122 People’s Republic of China; 3 Key Laboratory of Medical Cell Biology, Ministry of Education of China, China Medical University, Shenyang, 110122 People’s Republic of China; 4 Department of Pharmacology, Shenyang Medical College, Shenyang, 110034 People’s Republic of China; 5 Department of Neurosurgery, Shengjing Hospital of China Medical University, Shenyang, 110004 People’s Republic of China; 6 Liaoning Clinical Medical Research Center in Nervous System Disease, Shenyang, 110004 People’s Republic of China; 7 Key Laboratory of Neuro-oncology in Liaoning Province, Shenyang, 110004 People’s Republic of China

**Keywords:** MOV10, Circ-DICER1, miR-103a-3p, miR-382-5p, Gliomas, Angiogenesis

## Abstract

**Background:**

RNA binding proteins (RBPs) have been reported to interact with RNAs to regulate gene expression. Circular RNAs (circRNAs) are a type of endogenous non-coding RNAs, which involved in the angiogenesis of tumor. The purpose of this study is to elucidate the potential roles and molecular mechanisms of MOV10 and circ-DICER1 in regulating the angiogenesis of glioma-exposed endothelial cells (GECs).

**Methods:**

The expressions of circ-DICER1, miR-103a-3p and miR-382-5p were detected by real-time PCR. The expressions of MOV10, ZIC4, Hsp90 and PI3K/Akt were detected by real-time PCR or western blot. The binding ability of circ-SHKBP1 and miR-544a / miR-379, ZIC4 and miR-544a / miR-379 were analyzed with Dual-Luciferase Reporter System or RIP experiment. The direct effects of ZIC4 on the Hsp90β promoter were analyzed by the ChIP experiment. The cell viability, migration and tube formation in vitro were detected by CCK-8, Transwell assay and Matrigel tube formation assay. The angiogenesis in vivo was evaluated by Matrigel plug assay. Student’s t-test (two tailed) was used for comparisons between two groups. One-way analysis of variance (ANOVA) was used for multi-group comparisons followed by Bonferroni post-hoc analysis.

**Results:**

The expressions of RNA binding proteins MOV10, circ-DICER1, ZIC4, and Hsp90β were up-regulated in GECs, while miR103a-3p/miR-382-5p were down-regulated. MOV10 binding circ-DICER1 regulated the cell viability, migration, and tube formation of GECs. And the effects of both MOV10 and circ-DICER1 silencing were better than the effects of MOV10 or circ-DICER1 alone silencing. In addition, circ-DICER1 acts as a molecular sponge to adsorb miR-103a-3p / miR-382-5p and impair the negative regulation of miR-103a-3p / miR-382-5p on ZIC4 in GECs. Furthermore, ZIC4 up-regulates the expression of its downstream target Hsp90β, and Hsp90 promotes the cell viability, migration, and tube formation of GECs by activating PI3K/Akt signaling pathway.

**Conclusions:**

MOV10 / circ-DICER1 / miR-103a-3p (miR-382-5p) / ZIC4 pathway plays a vital role in regulating the angiogenesis of glioma. Our findings not only provides novel mechanisms for the angiogenesis of glioma, but also provide potential targets for anti-angiogenesis therapies of glioma.

**Electronic supplementary material:**

The online version of this article (10.1186/s13046-018-0990-1) contains supplementary material, which is available to authorized users.

## Background

Malignant gliomas are considered to be the most common intracranial primary cancers. The morbidity and mortality rates remains obstinately high [1]. At present, combined therapy of surgery and perfect radiotherapy or chemotherapeutics still unable to prolong the median survival of patients [2]. Angiogenesis is the basic pathological feature of malignant tumors, and plays vital roles in the development and progression of tumor [3]. Malignant gliomas have strong vascular invasiveness, and these abnormal blood vessels in structure and function are associated with the development and progression of tumor, which attributes the gene therapy of anti-tumor angiogenesis as one of the hot topics in recent years [4].

RNA-binding proteins (RBPs) play an important role in the regulation of post-transcription levels, including participation in RNA splicing, RNA transport, mRNA stabilization, polyadenosinaction, and intracellular localization [5]. RNA-binding proteins have become a research hotspot duo to their relationship with many human diseases [6]. Research demonstrates RNA-binding proteins can bind with non-coding RNAs and affect their functions [7]. Moloney leukemia virus 10 (MOV10) is a newly discovered RNA binding protein, belonging to the RNA helicase superfamily, and is a member of the RNA-induced silencing complex (RISC) [8]. And studies have found that mRNA and protein expressions of MOV10 in cancer cells such as cervical cancer are 2 to 3 folds higher than normal cells [9], which suggesting that MOV10 may be related to the development and progression of tumors. At present, the role of MOV10 has not been reported in the angiogenesis of glioma.

Circular RNAs (circRNAs) are a newly discovered class of non-coding RNAs, which are involved in the development and progression of many diseases [10]. Recent studies showed circRNAs are acted as the sponges of microRNAs (miRNAs) to relieve their inhibition in mRNA of target gene [11]. Besides, circRNAs can also bind with RNA-binding proteins to play the biological roles [12]. Dicer 1 is a kind of nucleic acid endonuclease, belonging to the RNase III family, and is an important molecule during the production of small interference RNAs (siRNAs) and miRNAs [13]. And researches have shown Dicer 1 is expressed differently in a variety of tumors and is related to the prognosis of tumors. For example, the prognosis of melanoma patient with high expression of Dicer 1 is poor [14] and ovarian cancer patients with low expression of Dicer1 have a poor prognosis [15]. There are no relevant reports between Dicer 1 and glioma currently.

MiRNAs are a class of endogenous non-coding RNAs composed of 18 to 23 nucleotides. MiRNAs bind with 3’UTR of target gene to inhibit the translation or degradation of target mRNA [16]. Aberrant expressions of miR-103a-3p are in a variety of tumors, such as high expression in osteosarcoma [17], but low expression in glioma stem cells and glioma tissue [18]. Research found that miR-103a-3p was an important molecule in the process of glioblastoma [19]. Besides, miR-103a-3p can promote the apoptosis of human colorectal adenocarcinoma associated endothelial cells and inhibit angiogenesis of tumors [20]. MiR-382-5p play an inhibitory effect in most tumors, such as non-small cell lung cancer and colon cancer [21, 22]. In gastric cancer, hypoxia causes high expression of miR-382 and promotes the proliferation, migration, and tubular formation of endothelial cells [23]. At present, the role of miR-103a-3p and miR-382-5p in glioma angiogenesis has not been reported.

Zinc finger of the cerebellum 4 (ZIC4) is a member of ZIC family. It has five highly conservative C2H2 series repeats that play an important role in neural development [24]. And ZIC4 is expressed in malignant glioma tissue and is highly expressed in meningeal tissue [25]. However, it has not been reported whether ZIC4 has a regulatory effect on angiogenesis of glioma.

Heat shock protein 90 (Hsp90) is an ATPase-directed molecular chaperone. It has two isoforms, Hsp90α and Hsp90β. Hsp90β plays an important role in the growth and proliferation of tumors [26]. Recent study have revealed that Hsp90β promoted endothelial cell-dependent tumor angiogenesis in hepatocellular carcinoma [27]. Moreover, studies have shown that Hsp90β is highly expressed in glioma tissue, and its inhibitors can inhibit the migration and invasion of glioma cells and inhibit the secretion of vascular endothelial growth factor [28]. However, the role of Hsp90β in angiogenesis of glioma have not been well unveiled.

In the present study, we first investigated the endogenous expressions and functions of MOV10, circ-DICER1, miR-103a-3p, miR-382-5p, ZIC4 and Hsp90β in GECs, and further clarified the possible regulating relationships of the above mentioned factors and their roles in the angiogenesis of GECs in vitro. It is aimed to provide potential targets for glioma treatment with regard to anti-angiogenesis.

## Methods

### Cells lines and culture

The immortalized human cerebral microvascular endothelial cell line hCMEC/D3 was gifted from Dr. Couraud (Institut Cochin, Paris, France). ECs applied in this study were within 30 passages. Cells were cultured in endothelial basal medium (EBM-2; Lonza, Walkersville, MD, USA) supplemented with 5% fetal bovine serum “Gold”, 1% penicillin-streptomycin, 1% chemically defined lipid concentrate, 1 ng/ml bFGF, 1.4 μM hydrocortisone, 5 μg/ml ascorbic acid and 10 mM HEPES. Human glioma U87MG and human embryonic kidney 293 T (HEK293T) cell lines were obtained from the Shanghai Institutes and they were cultured in Dulbecco’s modified Eagle’s medium (DMEM) of high glucose with 10% fetal bovine serum. Primary NHA were obtained from the ScienCell Research Laboratories (Carlsbad, CA) and cultured under the conditions instructed by the manufacturer. All cells were maintained in a humidified incubator at 37 °C with 5% CO_2_.

Glioma conditioned medium and astrocyte conditioned medium were collected from the indicated human glioma cells grown in 100-mm-diameter Petri dishes. Cells were washed twice with serum free medium when they grew to near confluency and incubated in serum free EBM-2 medium for 24 h. The supernatant was harvested, centrifuged at 2000 g at 4 °C for 10 min, and supplemented with 5% FBS, 1% penicillin-streptomycin, 1% chemically defined lipid concentrate, 1 ng/mL bFGF, 1.4 μM hydrocortisone, 5 μg/mL ascorbic acid and 10 mM HEPES, epidermal growth factor (EGF), and hydrocortisone prior to use. Astrocyte conditioned medium was used as a negative control (NC).

### Plasmid construction and cell transfection

Silencing plasmid of circ-DICER1 was constructed in pGPU6/Hygro vector (Genechem Co, Shanghai, China). And a non-targeting sequence was used as a NC. Overexpression plasmid of Hsp90β (NM_003299.2) with pIRES2-EGFP (GenScript, Piscataway, NJ, USA) and silencing plasmids of MOV10 (NM_001130079.2) and Hsp90β with pGPU6/GFP/Neo (GenePharma, Shanghai, China) were constructed, respectively. An empty vector was used as a blank control. ZIC4 (NM_001168378.1) full length (with 3′-UTR) plasmid, ZIC4 (without 3′-UTR) plasmid (GenScript, Piscataway, NJ, USA), short-hairpin ZIC4 plasmid (GenePharma, Shanghai, China) and their respective non-targeting sequence (negative control, NC) were constructed. ECs were seeded in 24-well plates and transfected using LTX and Plus reagents (Life Technologies) when they were at 70 ~ 80% confluence. Stable cell lines were established via Geneticin (G418; Sigma-Aldrich, StLouis, MO, USA) and Hygromycin (Solarbio, China) selection. We selected the G418 or Hygromycin resistant clones after 3 ~ 4 weeks.

For transient transfection assays, miR-103-3p/miR-382-5p agomir (miR-103-3p/miR-382-5p (+)), miR-103-3p/miR-382-5p antagomir (miR-103-3p/miR-382-5p (−)), and their NC sequence (miR-103-3p/miR-382-5p (+) NC and miR-103-3p/miR-382-5p (−)NC) were synthesized (GenePharma, Shanghai, China) and transiently transfected into GECs using Opti-MEM and Lipofectamine 3000 reagents (Life Technologies Corporation, Carlsbad, CA, USA), respectively. Cells were collected 48 h after transfection. Sequences of shcirc-DICER1, shMOV10, shZIC4, shHsp90β and shNC were shown in Table 1.

**Table 1:** Sequences of shRNA template

Gene		Sequence(5′- > 3′)
MOV10	Sence	CACCGCAACAGCCCATCCTTCTTCATTCAAGAGATGAAGAAGGATGGGCTGTTGCTTTTTTG
	Antisence	GATCCAAAAAAGCAACAGCCCATCCTTCTTCATCTCTTGAATGAAGAAGGATGGGCTGTTGC
Circ-DICER1	Sence	CCGGCGTCTGTTCAGTTCTCATTATCTCGAGATAATGAGAACTGAACAGACGTTTTTG
	Antisence	AATTCAAAAACGTCTGTTCAGTTCTCATTATCTCGAGATAATGAGAACTGAACAGACG
ZIC4	Sence	CACCGGGAAGGTCTTTGCTAGATCATTCAAGAGATGATCTAGCAAAGACCTTCCCTTTTTTG
	Antisence	GATCCAAAAAAGGGAAGGTCTTTGCTAGATCATCTCTTGAATGATCTAGCAAAGACCTTCCC
Hsp90β	Sence	CACCGCCCATGGAGGAAGAAGAAGCTTCAAGAGAGCTTCTTCTTCCTCCATGGGCTTTTTTG
	Antisence	GATCCAAAAAAGCCCATGGAGGAAGAAGAAGCTCTCTTGAAGCTTCTTCTTCCTCCATGGGC
NC	Sence	CACCGTTCTCCGAACGTGTCACGTCAAGAGATTACGTGACACGTTCGGAGAATTTTTTG
	Antisence	GATCCAAAAAATTCTCCGAACGTGTCACGTAATCTCTTGACGTGACACGTTCGGAGAAC
MOV10	Sence	CACCGCAACAGCCCATCCTTCTTCATTCAAGAGATGAAGAAGGATGGGCTGTTGCTTTTTTG
	Antisence	GATCCAAAAAAGCAACAGCCCATCCTTCTTCATCTCTTGAATGAAGAAGGATGGGCTGTTGC

### Quantitative real-time PCR (qRT-PCR)

Total RNA was extracted from the cultured cells with Trizol reagent as described by the manufacturer (Life Technologies Corporation, Carlsbad, CA, USA). One Step PrimeScript™ RT-PCR Kits (Takara, RR064A, Japan) were used for measurement of circ-DICER1. In addition, RNase-R was used to confirm the existence of circ-DICER1, and eliminated the influence of liner RNAs. TaqMan MicroRNA Reverse Transcription kit and Taqman Universal Master Mix II (Applied Biosystems) were used to quantify miR-103-3p, miR-382-5p and U6 expression. One Step SYBR® PrimeScript™ RT-PCR Kit (Takara Biomedical Technology, Dalian, China) was used for the detections of MOV10, ZIC4, Hsp90β and GAPDH. Their expressions were normalized to endogenous control GAPDH and fold change was determined as 2^−ΔΔCt^ in gene expression. For details, see Table 2.

**Table 2:** Primers and probes used for RT-qPCR

Primer or Probe	Gene	Sequence (5′- > 3′) or Assay ID
Primer	circ-DICER1	F: TGTAATAATTGTAGCCAAGTAAATCTCC
		R: AAGTAACCATTTTTCAAAACATTCAAG
		P:FAM + TAAAGTTATCGTCTGTTCAGTTCTCATTATGACTTG+BHQ1
	GAPDH	F:GGACCTGACCTGCCGTCTAG
		R:TAGCCCAGGATGCCCTTGAG
		P:FAM + CCTCCGACGCCTGCTTCACCACCT+Eclipse
	MOV10	F: CCATGAGGCACATTGTTACG
		R:AAGTGCTTCACCACCTGCTT
	ZIC4	F:CTAGCGACAAGCCATACACG
		R:GTAGCCGAATCGTAGCCAGA
	Hsp90β	F:GTGGGTTCAGATGAGGAGGA
		R:TCTGGTCCAAATAGGCTTGG
Probe	MiR-103a-3p	000439(Applied biosystems)
	MiR-382-5p	000572(Applied biosystems)
	U6	001973(Applied biosystems)

### Cell viability assay

Endothelial cells (ECs) viability was determined by the Cell Counting kit-8 (CCK-8) assay (Beyotime Institute of Biotechnology, Jiangsu, China). Cells were seeded in 96-well plates in triplicate and incubated in glioma conditioned medium for 24 h, respectively. Each well was incubated with 10 μl CCK-8 for 2 h and the absorbance was measured at 450 nm using a spectrophotometer (Molecular Devices, United States).

### Cell migration assay

ECs migration in vitro was assayed using a Transwell chamber (Costar, Corning, NY, USA) with a polycarbonic membrane (6.5 mm in diameter and 8 μm pore size). ECs were suspended into single cells in serum-free medium at the density of 5 × 10 [5] cells/ml. 100 μl suspension was added to the upper compartment and 600 μl of glioma conditioned medium supplemented with 10% FBS was added into the lower chamber. Cells were incubated for 48 h at 37 °C. Non-migrating cells on the top surface of membrane were removed with cotton swabs. Cells that migrated to the lower surface of the membrane were fixed with 3:1 methanol:glacial acetic acid, stained with 10% Giemsa solution for 30 min at 37 °C, and washed twice with phosphate buffer saline (PBS).Then the pictures of stained cells were taken with an inverted microscope. Then, stained cells in five randomly fields were randomly chosen for statistics.

### Tube formation assay

Matrigel assay was used to evaluate in vitro angiogenesis activity by quantifying tube formation as previously described. In total, 96-well culture plates were coated with 100 μL Matrigel (BD Biosciences, Bedford, MA, USA) per well and then allowed to polymerize for 30 min at 37 °C. Then, cells were resuspended in 100 μl glioma conditioned medium at a density of 4 × 10 [5] cells per ml and added to Matrigel-coated wells. After maintained in 37 °C for 24 h, pictures of each culture were taken with a digital camera system (Olympus, Tokyo, Japan) and total tubule length and number of branches were were measured using Chemi Imager 5500 V2.03 software.

### Western blot assay

Total proteins from the cells were lysed with ice-cold RIPA buffer with protease inhibitors (Beyotime Institute of Biotechnology). The protein concentration of the sample was determined with the BCA protein assay kit (Beyotime Institute of Biotechnology, Jiangsu, China). Electrophoresis was conducted to equal amount of protein samples (40 μg) with SDS–polyacrylamide gel electrophoresis and then transferred to PVDF membranes. Membranes were incubated in 5% fat-free milk in TBST and then incubated with primary antibodies against MOV10 (1:500, Proteintech, Chicago, IL, USA), ZIC4 (1:1000, Santa Cruz Biotechnology), Hsp90β (1:200; Abcam, USA), p-PI3K (1:500, Bioworld, Minneapolis, MN, United States), PI3K (1:1000, CST, EUGENE), p-AKT (1:2000, CST, EUGENE), AKT (1:2000, CST, EUGENE), and GAPDH (1:1000, Proteintech, Chicago, IL, United States).

### Reporter vector construction and luciferase reporter assay

The potential binding sequence of miR-103-3p/miR-382-5p in circ-DICER1 gene and its mutant sequence was amplified by PCR, synthesized and cloned into the pmirGLO dual-luciferase vector (Promega, Madison, WI, USA). Wild-type pmirGLO- circ-DICER1 (or circ-DICER1 mutant) reporter plasmid and miR-103-3p/miR-382-5p agomir (or agomir NC) were co-transfected into HEK293T cells. The pmirGLO empty vector was transfected as “Control” group. Luciferase activity was measured 48 h after transfection through the Dual-Luciferase Reporter System (Promega). The renilla luciferase activity was used as internal control to normalize the value. The relative luciferase activity was expressed as the ratio of firefly luciferase activity to renilla luciferase activity. Wild-type ZIC4–3′UTR reporter plasmid (ZIC4-wt) and mutated-type ZIC4–3′UTR reporter plasmid (ZIC4-mut) were constructed with pmirGLO-promoter vector. Following transfection approach and measurement of luciferase activities were performed as described above.

### RNA-binding protein immunoprecipitation (RIP) assay

Cells were lysed in complete RNA lysis buffer containing magnetic beads conjugated with human anti-MOV10, anti-MOV10 antibody or negative control normal mouse IgG. And whole cell lysate of the control groups and anti-miR-103a-3p/anti-miR-382-5p groups were incubated with RIP immunoprecipitation buffer containing magnetic beads conjugated with human anti-Argonaute2 (Ago2) antibody (Millipore), and NC normal mouse IgG (Millipore). The samples were incubated with Proteinase K and then immunoprecipitated RNA was isolated. Purified RNA was obtained and then applied to quantitative PCR with reverse transcription analysis.

### Chromatin immunoprecipitation (ChIP) assay

ChIP assay was performed with Simple ChIP Enzymatic Chromatin IP Kit (Cell signaling Technology, Danvers, Massachusetts, USA) according to the manufacturer’s protocol. Briefly, cells were crosslinked with EBM-2 containing 1% formaldehyde and collected in lysis buffer. 2% aliquots of lysates were used as an input control and the remaining lysates were immunoprecipitated with normal rabbit IgG or ZIC4 antibody (Santa Cruz Biotechnology) followed by immunoprecipitation with Protein G Agarose Beads in each sample during an overnight incubation at 4 °C with gentle shaking. Then the DNA crosslink was reversed by 5 mol/L NaCl and Proteinase K and finally DNA was purified. Immunoprecipitated DNA was amplified by PCR using primers, which were listed in Table 3.

**Table 3:** Primers used for ChIP experiments

Gene	Binding site or Control	Sequence (5′- > 3′)	Product size (bp)	Annealing temperature (°C)
Hsp90β	PCR1	F: CACGCCCGGCTAATTTTTGT	203	60.1
		R: TCCAGCCTGGGCAACAAAA		
	PCR2	F: TTAAAATTGGCCAGGCGCAG	101	59.8
		R: CCAGGCTGGTTTCGAACTCC		

### In vivo Matrigel plug assay

Matrigel plug assay was conducted to measure the angiogenesis as previously described [29]. Nude mice were purchased from the National Laboratory Animal Center (Beijing, China). Four-week-old BALB/c athymic nude mice were fed with autoclaved food and water during the experiment. All the experiments with nude mice were performed strictly in accordance with the protocol approved by the Administrative Panel on Laboratory Animal Care of Shengjing Hospital. In brief, GECs re-suspended in 400 mL of solution containing 80% Matrigel at a density of 3 × 10 [5] were subcutaneously injected. After 4 days, plugs were removed, weighed, photographed, and collected by dissolving in 400 μm PBS (overnight incubation at 4 °C). The content of hemoglobin (Sigma) was determined by Drabkin’s solution (Sigma) according to manufacturer’s directions.

### Statistical analysis

Experimental data were presented as mean ± standard deviation (SD) from at least three independent experiments. All statistical analyses were performed with GraphPad Prism 5 (GraphPad Software, La Jolla, CA, USA). Student’s t-test (two tailed) was used for comparisons between two groups. One-way analysis of variance (ANOVA) was used for multi-group comparisons followed by Bonferroni post-hoc analysis. The value of *p* < 0.05 was considered statistically significant.

## Results

### MOV10 was up-regulated in GECs, knockdown of MOV10 inhibited the angiogenesis of GECs in vitro, and MOV10 combined with circ-DICER1 and regulated its expression

**Fig. 1:**
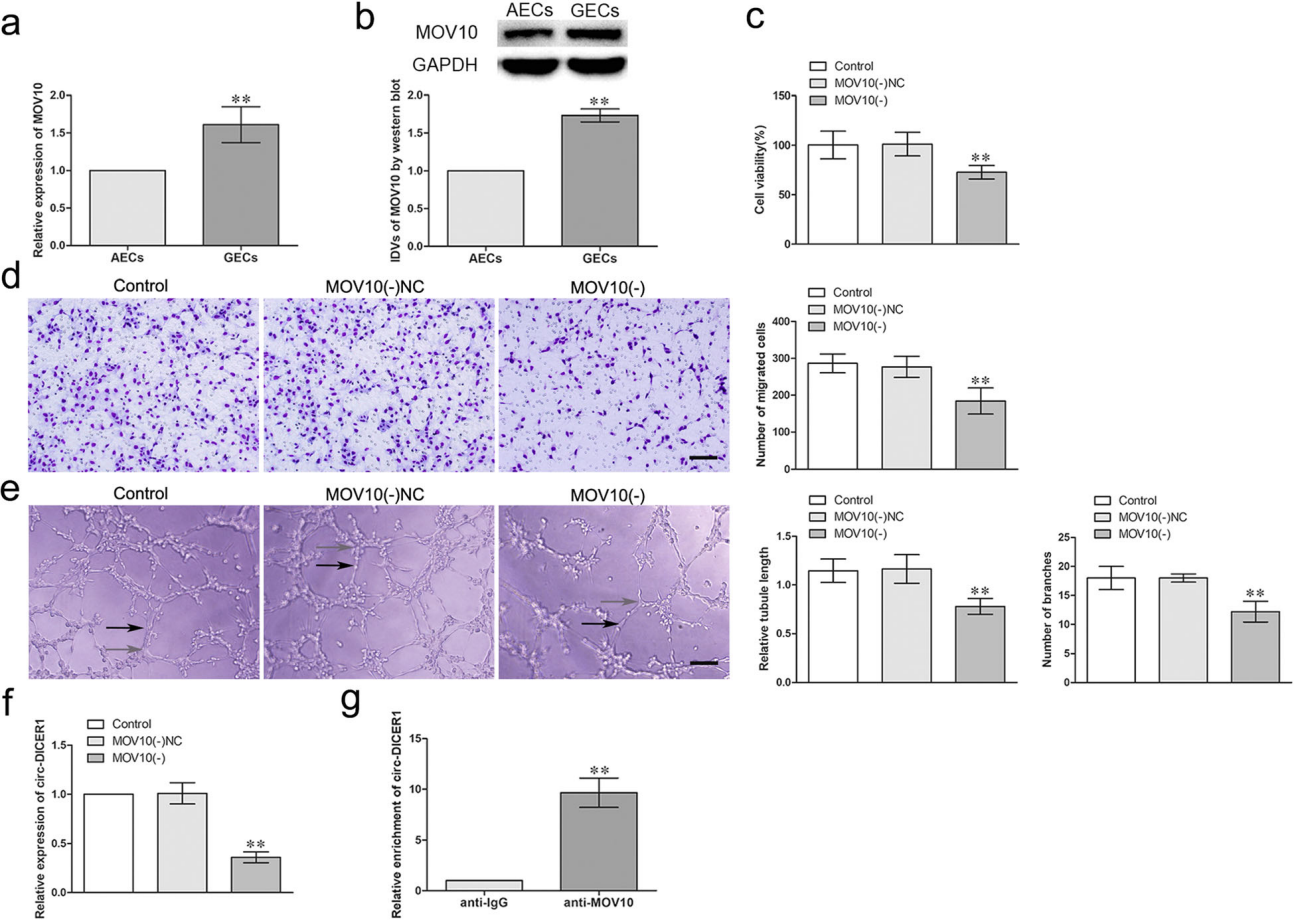
The expressions of MOV10 in GECs and knockdown of MOV10 suppressed the viability, migration and tube formation of GECs in vitro and MOV10 combined with circ-DICER1 and regulated its expression. **a-b** The mRNA and protein expressions of MOV10 in AECs and GECs were evaluated by qRT-PCR and western blot. GAPDH was used as an endogenous control. IDVs represents “Integrated Density Values”. Data represent means ± SD (*n* = 5, each group). **P < 0.01 vs. AECs group. **c** Effects of MOV10 knockdown on cell viability of GECs were detected by CCK-8 assay. Data are presented as the means ± SD (*n* = 5, each group). **P < 0.01 vs MOV10 (−) NC group. **d** Effects of MOV10 knockdown on migration of GECs were detected by Transwell assay. Data are presented as the means ± SD (*n* = 5, each group). **P < 0.01 vs MOV10 (−) NC group. Scale bar represents 30 μm. **e** Effects of MOV10 knockdown on tube formation of GECs were measured by Matrigel tube formation assay. Data are presented as the means ± SD (*n* = 5, each group). **P < 0.01 vs MOV10 (−) NC group. Scale bar represents 30 μm. **f** Effects of MOV10 knockdown on expression of circ-DICER1 were detected by qRT-PCR. Data are presented as the means ± SD (*n* = 5, each group). **P < 0.01 vs MOV10 (−) NC group. **g** Relative enrichment of circ-DICER1 in anti-IgG and anti-MOV10 were detected by RNA immunoprecipitation assay. Data represent means ± SD (*n* = 3, each group). **P < 0.01 vs. anti-IgG group

The RNA binding protein gene expression profiles were analyzed in astrocyte-exposed endothelial cells (AECs) and U87 glioma-exposed endothelial cells (GECs). And MOV10 was found the most significantly up-regulated in GECs compared with that in AECs (Additional file 1: Figure S1A). The mRNA and protein expressions of MOV10 was further detected in AECs and GECs by qRT-PCR and western blot respectively. As shown in Fig. 1a, b, the mRNA and protein expressions of MOV10 were up-regulated (1.612 ± 0.2384-fold and 1.733 ± 0.0851 -fold) in GECs compared with that in AECs (*p* < 0.01). To assess the potential role in angiogenesis, MOV10 was stably knocked down in GECs and the transfection efficiency was validated by western blot (Additional file 1: Figure S1B). The effects of MOV10 knockdown on the viability, migration and tube formation of GECs were detected by CCK-8, Transwell and Matrigel tube formation assay. As shown in Fig. 1c-e, there was no significant difference in the viability, migration and tube formation of GECs between Control group and MOV10 (−) NC groups. However, the viability, migration and tube formation of GECs in MOV10 (−) group were significantly inhibited compared with MOV10 (−) NC group (*P* < 0.01). Furthermore, circular RNA gene expression profiles demonstrated circ-DICER1 (has-circ-0033079) was the most significantly down-regulated after MOV10 knockdown (Additional file 1: Figure S1C). As shown in Fig. 1f, there was no significant difference in the expression of circ-DICER1 between Control group and MOV10 (−) NC group. Nevertheless, the expression of circ-DICER1 was significantly decreased in MOV10 (−) group compared with MOV10 (−) NC group (*P* < 0.01). Furthermore, RNA-binding protein immunoprecipitation (RIP) assay was conducted to confirm the combination of MOV10 and circ-DICER1, The relative abundance of circ-DICER1 was increased in anti-MOV10 group compared with anti-IgG group (*P* < 0.01, Fig. 1g).

### Circ-DICER1 was up-regulated in GECs and knockdown of circ-DICER1 inhibited the viability, migration and tube formation of GECs

**Fig. 2:**
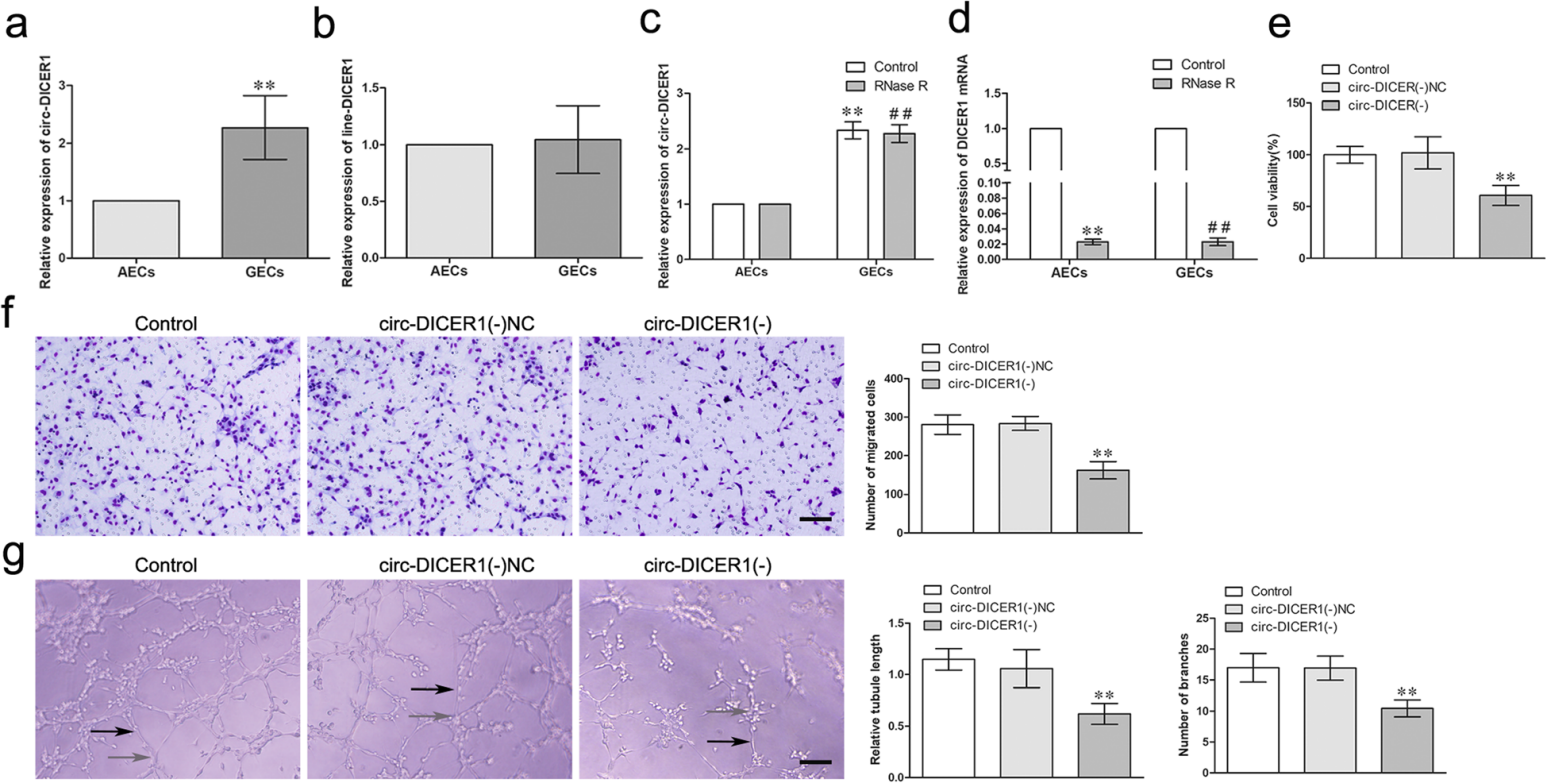
Circ-DICER1 was up-regulated in GECs and knockdown of circ-DICER1 suppressed the viability, migration and tube formation of GECs in vitro. **a** The relative expression of circ-DICER1 in AECs and GECs was detected by qRT-PCR. Data represent means ± SD (*n* = 5, each group). **P < 0.01 vs. AECs group. **b** The relative expression of DICER1 was detected in AECs and GECs by qRT-PCR. GAPDH was used as an endogenous control. Data represent means ± SD (*n* = 5, each group). **c** The expression of circ-DICER1 in GECs with RNase R treatment. Data represent means ± SD (*n* = 5, each group). **P < 0.01 versus. Control group in AECs; ^##^*P* < 0.01 versus. RNase R group in AECs. **d** The mRNA expression of DICER1 in GECs with RNase R treatment. Data represent means ± SD (*n* = 5, each group). **P < 0.01 versus. Control group in AECs; ^##^*P* < 0.01 versus. Control group in GECs. **e** Effects of circ-DICER1 knockdown on the cell viability of GECs were detected by CCK-8 assay. Data are presented as the means ± SD (*n* = 5, each group). **P < 0.01 vs knockdown of circ-DICER1 NC group. **f** Effects of circ-DICER1 knockdown on the migration of GECs were detected by Transwell assay. Data are presented as the means ± SD (*n* = 5, each group). **P < 0.01 vs knockdown of circ-DICER1 NC group. Scale bar represents 30 μm. **g** Effects of circ-DICER1 knockdown on tube formation of GECs were measured by Matrigel tube formation assay. Data are presented as the means ± SD (*n* = 5, each group). **P < 0.01 vs knockdown of circ-DICER1 NC group. Scale bar represents 30 μm

The expression of circ-DICER1 in GECs and AECs was detected by qRT-PCR. As shown in Fig. 2a, the expression of circ-DICER1 was significantly up-regulated (2.272 ± 0.5525-fold) in GECs compared with AECs (*P* < 0.01). However, there was no significant difference of linear DICER1 between GECs and AECs (Fig. 2b). Furthermore, RNase R, an RNA exonuclease that degrades linear RNAs but does not degrade circular forms, was used to confirm the circular form RNA. As expected, circ-DICER1 was resistant to RNase R treatment, while linear DICER1 was significantly reduced in cells treated with RNase R (Fig 2. c-d). Then the shRNA of circ-DICER1 was transfected into GECs to knock down circ-DICER1 to further explore the potential role of circ-DICER1 in GECs, and the transfection efficiency was shown in Additional file 2: Figure S2A. In addition, the expression of DICER1 was detected after knockdown of circ-DICER1 to confirm the circular form instead of linear form of DICER1 was inhibited. As shown in Additional file 2: Figure S2B, no significant change of DICER1 expression between the knockdown of circ-DICER1 group and the knockdown of circ-DICER1 NC group. Meanwhile, DICER1 was knocked down to detect whether sh-DICER1 influenced circ-DICER1 expression. Transfection efficiency of DICER1 was verified (Additional file 2: Figure S2C), also no significant change of circ-DICER1 expression between the sh-DICER1 group and short hairpin (sh)-NC group (Additional file 2: Figure S2D). Furthermore, circ-DICER1 knockdown attenuated the viability, migration and tube formation of GECs (*P* < 0.01, Fig. 2e-g). These data suggested that circ-DICER1 knockdown impaired GECs angiogenesis.

### MiR-103a-3p/miR-382-5p functionally targeted circ-DICER1, and reversed the circ-DICER1-mediated angiogenesis of GECs

**Fig. 3:**
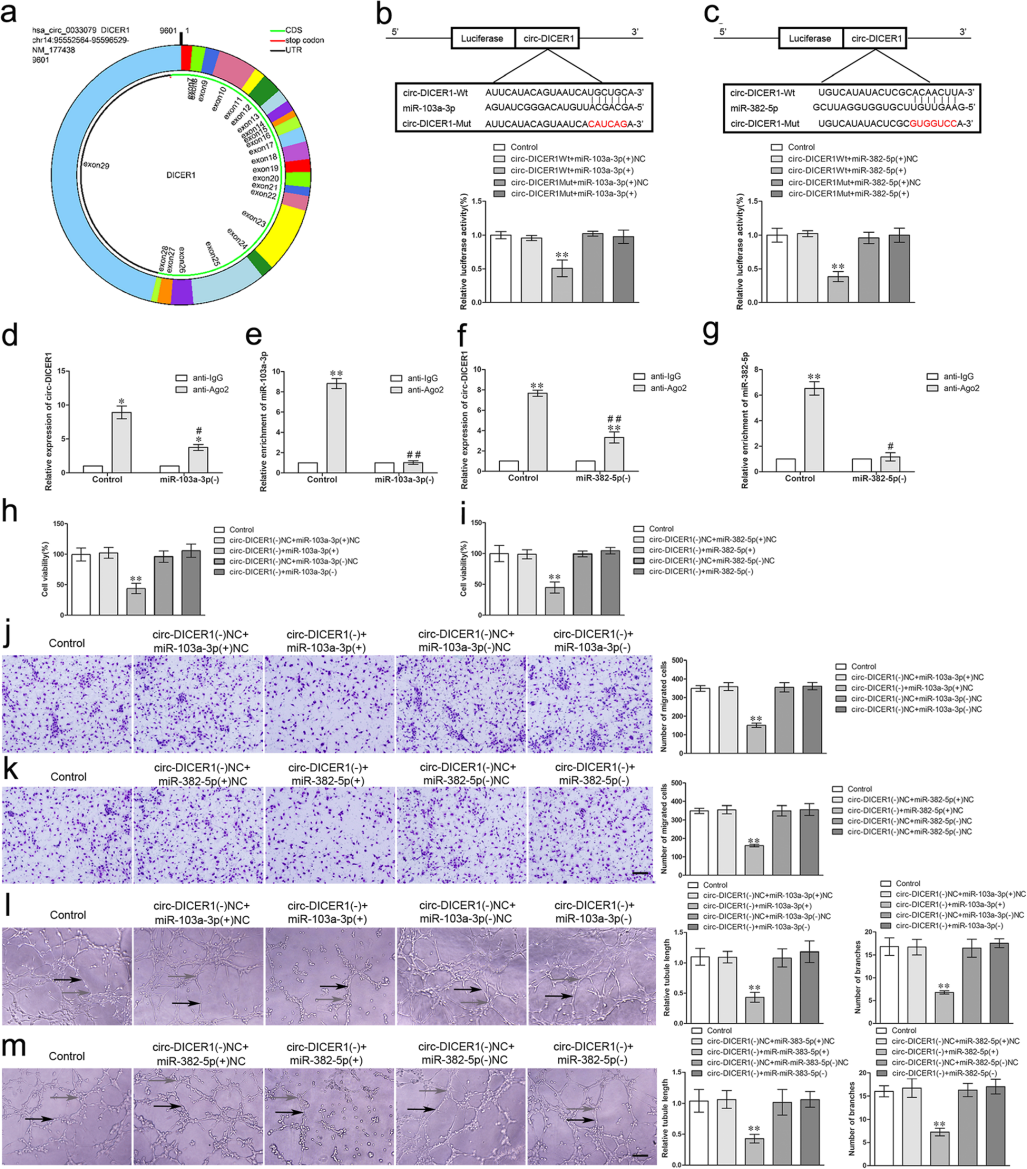
MiR-103a-3p/miR-382-5p functionally targeted circ-DICER1, and reversed the circ-DICER1-mediated viability, migration and tube formation of GECs. **a** Cartoon of circ-DICER1 arose from DICER1 gene by scanning DICER1 genomic DNA and circBase. **b-c** The putative binding sites between circ-DICER1 and miR-103a-3p/miR-382-5p were predicted and the relative luciferase activity was expressed as firefly/renilla luciferase activity. Values are means ± SD (*n* = 5, each group). **P < 0.01 vs. circ-DICER1 Wt + miR-103a-3p/miR-382-5p (+) NC group. **d-g** MiR-103a-3p/miR-382-5p was identified in circ-DICER1-RISC complex. Relative expression of circ-DICER1 and miR-103a-3p/miR-382-5p were measured using qRT-PCR. Data represent means ± SD (*n* = 5, each group). *P < 0.05, **P < 0.01 vs. anti-IgG group, ^#^*P* < 0.05, ^##^*P* < 0.01 vs. anti-Ago2 in control group. **h**-**i** The co-effects of circ-DICER1 and miR-103a-3p/miR-382-5p on the viability of GECs were evaluated by CCK-8 assay. Data are presented as the means ± SD (*n* = 5, each group). **P < 0.01 vs circ-DICER1 (−) NC + miR-103a-3p/miR-382-5p (+) NC group. **j**-**k** The co-effects of circ-DICER1 and miR-103a-3p/miR-382-5p on the migration of GECs were evaluated by Transwell assay. Data are presented as the means ± SD (*n* = 5, each group). **P < 0.01 vs circ-DICER1 (−) NC + miR-103a-3p/miR-382-5p (+) NC group. Scale bar represents 30 μm. **l**-**m** The co-effects of circ-DICER1 and miR-103a-3p/miR-382-5p on the tube formation of GECs were evaluated by Matrigel tube formation assay. Data are presented as the means ± SD (*n* = 5, each group). **P < 0.01 vs circ-DICER1 (−) NC + miR-103a-3p/miR-382-5p (+) NC group. Scale bar represents 30 μm

By scanning DICER1 genome and circBase (http://www.circbase.org), we found that circ-DICER1 was composed of exons from exon 7 to exon 28 (Fig. 3a). As reported, circRNAs could function as miRNAs sponge, and miRNAs could target circRNAs in a sequence-specific manner. Then the bioinformatics database (Starbase) suggested that there was a putative binding site between circ-DICER1 and miR-103a-3p/miR-382-5p, and miRNA gene expression profiles showed that miR-103a-3p and miR-382-5p were significantly down-regulated after circ-DICER1 knockdown (Additional file 2: Figure S2E). To confirm the hypothesis, we performed the dual luciferase reporter assay and RNA immunoprecipitation (RIP) assay. As expected, the relative luciferase activity was markedly suppressed in the circ-DICER1 wild-type (WT) + miR-103a-3p (+) group compared with that in the circ-DICER1 WT + miR-103a-3p (+) NC group. Nevertheless, there was no significant difference between the circ-DICER1 mutant (Mut) + miR-103a-3p (+) group and the circ-DICER1 Mut + miR-103a-3p (+) NC group (Fig. 3b). Additionally, similar results were achieved between circ-DICER1 and miR-382-5p (Fig. 3c). Meanwhile, a potential target was identified in linear DICER1 with miR-103a-3p. As shown in Additional file 2: Figure S2F, the relative luciferase activity was significantly reduced in the DICER1 WT + miR-103a-3p (+) group. RIP assay using antibody against Ago2 showed higher circ-DICER1 and miR-103a-3p levels in the Ago2 precipitates compared with the IgG precipitates. Moreover, knockdown of miR-103a-3p decreased the enrichment of circ-DICER1 and miR-103a-3p in Ago2 precipitates (Fig. 3d, e). Similar results were obtained in the relative abundance of circ-DICER1 and miR-382-5p (Fig. 3f, g). The above data indicated that circ-DICER1 sponged miR-103a-3p/miR-382-5p in a sequence-dependent manner.

To further clarify whether miR-103a-3p/miR-382-5p was involved in circ-DICER1-mediated angiogenesis of GECs, GECs with stable knockdown of circ-DICER1 were transiently transfected with miR-103a-3p/miR-382-5p agomir or antagomir. The cell viability (Fig. 3h, i), migration (Fig. 3j, k), and tube formation (Fig. 3l. m) of GECs in the circ-DICER1 (−) + miR-103a-3p/miR-382-5p (+) group were significantly decreased compared with those in the circ-DICER1 (−) NC + miR-103a-3p/miR-382-5p (+) NC group, which indicated miR-103a-3p/miR-382-5p reversed circ-DICER1-mediated inhibition of viability, migration, and tube formation of GECs.

### Overexpression of miR-103a-3p and miR-382-5p inhibited the angiogenesis of GECs in vitro

**Fig. 4:**
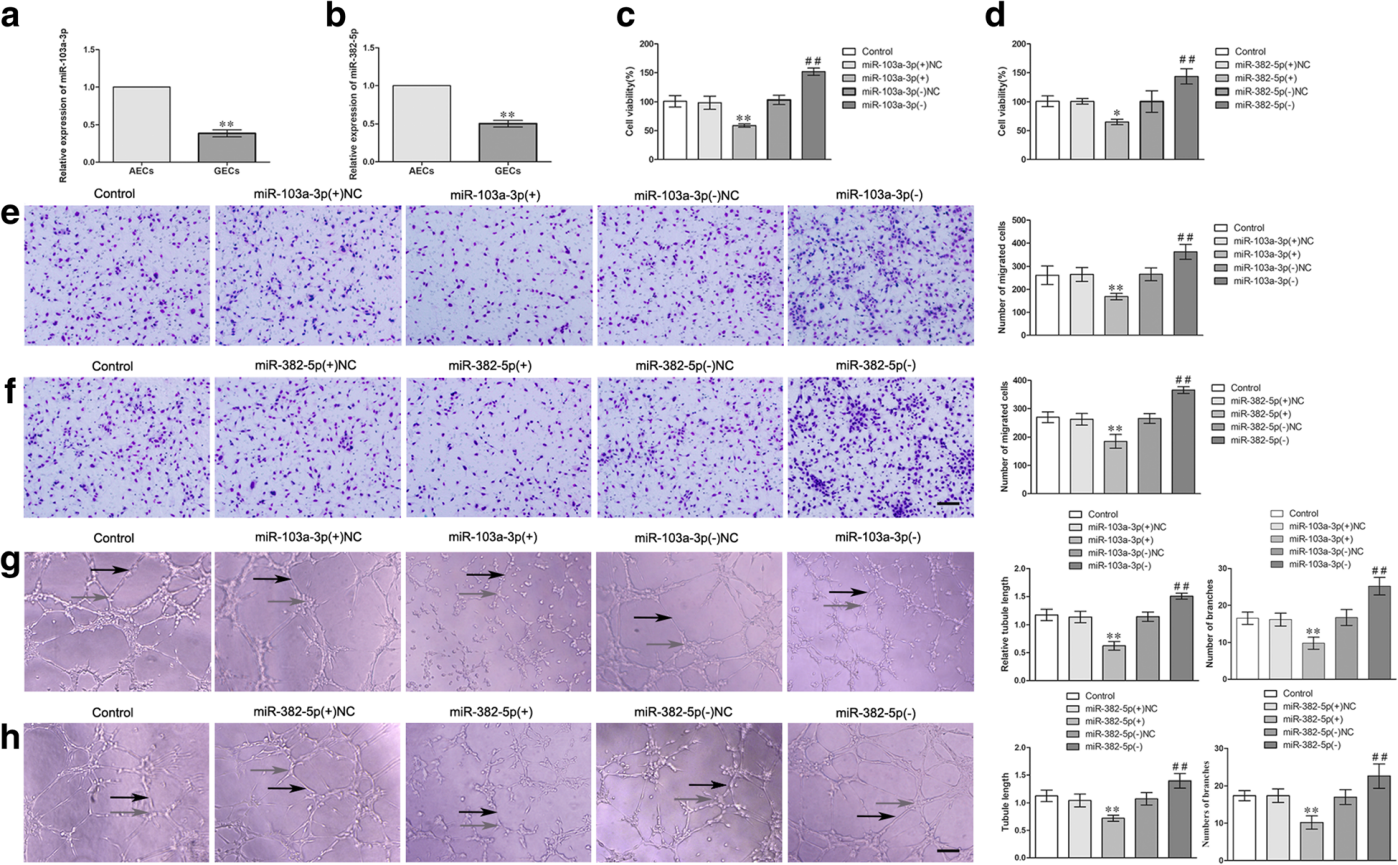
The relative expression of miR-103a-3p/miR-382-5p in GECs and miR-103a-3p/miR-382-5p regulated the viability, migration and tube formation of GECs. **a**-**b** The relative expression of miR-103a-3p/miR-382-5p in AECs and GECs were detected by qRT-PCR. U6 was used as an inner control. Data represent means ± SD (*n* = 3, each group). **P < 0.01 vs. AECs group. **c**-**d** The effects of miR-103a-3p/miR-382-5p on the viability of GECs were determined by CCK-8 assay. Values are means ± SD (*n* = 5, each group). *P < 0.05, **P < 0.01 vs. miR-103a-3p/miR-382-5p (+) NC group; ^##^*P* < 0.01 vs. miR-103a-3p/miR-382-5p (−) NC group. **e**-**f** The effects of miR-103a-3p/miR-382-5p on the migration of GECs by Transwell assay. Values are means ± SD (*n* = 5, each group). **P < 0.01 vs. miR-103a-3p/miR-382-5p (+) NC group; ^##^*P* < 0.01 vs. miR-103a-3p/miR-382-5p (−) NC group. Scale bar represents 30 μm. **g**-**h** The effects of miR-103a-3p/miR-382-5p on the tube formation of GECs were evaluated by Matrigel tube formation assay. Values are means ± SD (*n* = 5, each group). **P < 0.01 vs. miR-103a-3p/miR-382-5p (+) NC group; ^##^*P* < 0.01 vs. miR-103a-3p/miR-382-5p (−) NC group. Scale bar represents 30 μm

As shown in Fig. 4a, b, the endogenous expressions of miR-103a-3p and miR-382-5p were significantly down-regulated (0.3847 ± 0.0470-fold and 0.5029 ± 0.0439) in GECs compared with AECs (*P* < 0.01). Overexpression or silencing of miR-103a-3p/miR-382-5p was performed to further understand their role in the angiogenesis of GECs, and the transfection efficiency was evaluated by qRT-PCR (Additional file 2: Figure S2G-H). Overexpression of miR-103a-3p/miR-382-5p inhibited the cell viability (Fig. 4c, d), migration (Fig. 4e, f), and tube formation (Fig. 4g, h) of GECs, whereas silencing of miR-103a-3p/miR-382-5p produced the opposite results (Fig. 4c-h). These data indicated that overexpression of miR-103a-3p/miR-382-5p significantly inhibited the angiogenesis of GECs.

### ZIC4 was a target of miR-103a-3p/miR-382-5p, and was involved in circ-DICER1 and miR-103a-3p/miR-382-5p-mediated angiogenesis of GECs

**Fig. 5:**
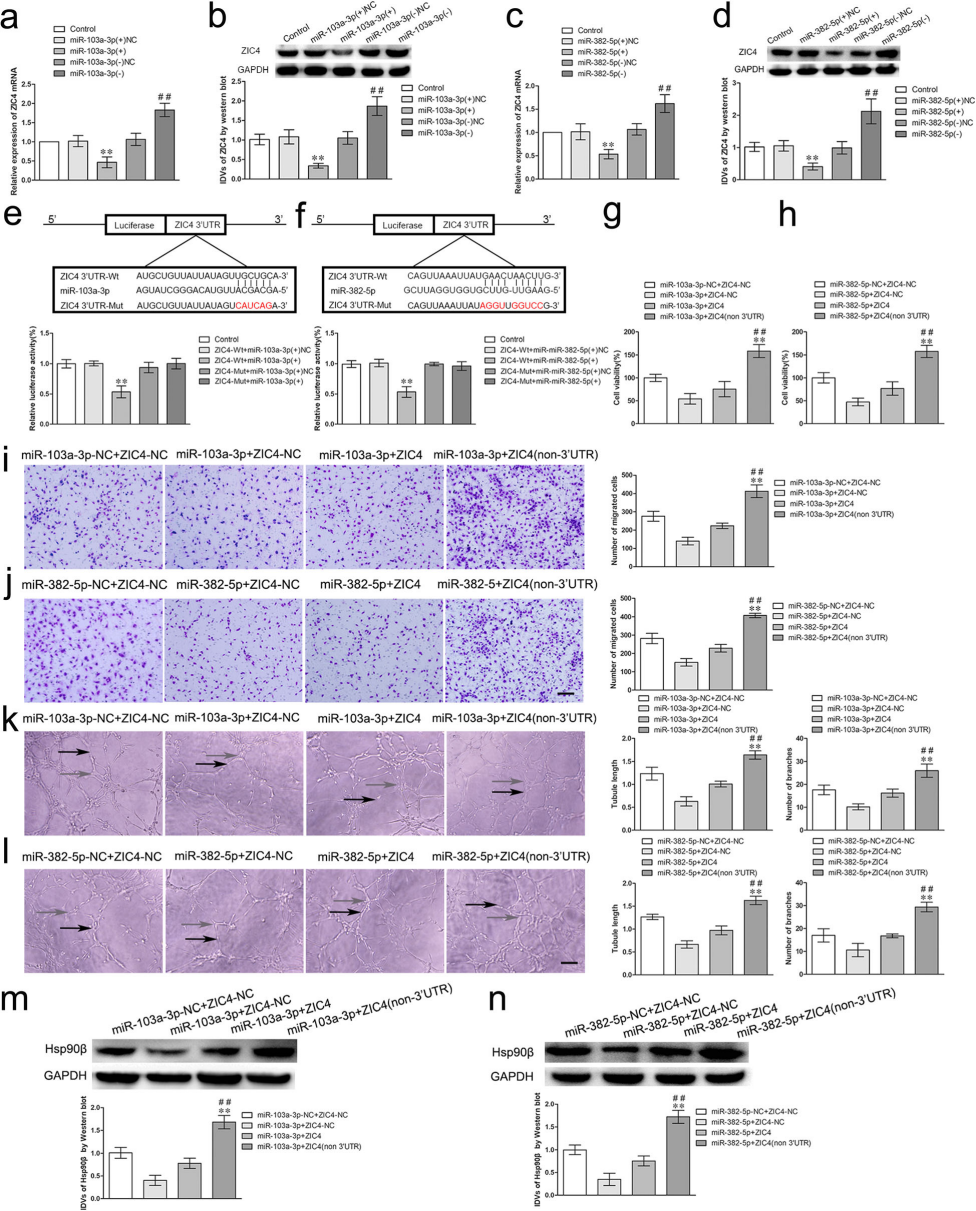
miR-103a-3p/miR-382-5p regulated the viability, migration and tube formation of GECs and Hsp90β expression by targeting ZIC4 3’-UTR. **a**-**d** The mRNA and protein expressions of ZIC4 were regulated by miR-103a-3p/miR-382-5p. Values represent the means ± SD (*n* = 5, each group). **P < 0.01 vs. miR-103a-3p/miR-382-5p (+) NC group. ^##^*P* < 0.01 vs. miR-103a-3p/miR-382-5 (−) NC group. **e**-**f** The putative binding sites between ZIC4 and miR-103a-3p/miR-382-5p were predicted respectively and the relative luciferase activity was expressed as firefly/renilla luciferase activity. Values are means ± SD (*n* = 5, each group). **P < 0.01 vs. ZIC4 Wt + miR-103a-3p/miR-382-5p (+) NC group. **g**-**h** The co-effects of miR-103a-3p/miR-382-5p and ZIC4 on the viability of GECs was evaluated by CCK8 assay. Data are presented as the means ± SD (*n* = 5, each group), **P < 0.01 vs. miR-103-3p / miR-382-5p + ZIC4 group; ^##^*P* < 0.01 vs. miR-103-3p/miR-382-5p + ZIC4-NC group. **i**-**j** The co-effects of miR-103a-3p/miR-382-5p and ZIC4 on the migration of GECs were evaluated by Transwell assay. Data are presented as the means ± SD (*n* = 5, each group), **P < 0.01 vs. miR-103-3p / miR-382-5p + ZIC4 group; ^##^*P* < 0.01 vs. miR-103-3p/miR-382-5p + ZIC4-NC group. **k**-**l** The co-effects of miR-103a-3p/miR-382-5p and ZIC4 on the tube formation of GECs were evaluated by Matrigel tube formation assay (black arrows, tube structures; gray arrows, tube branches). Data are presented as the means ± SD (*n* = 5, each group), **P < 0.01 vs. miR-103-3p / miR-382-5p + ZIC4 group; ^##^*P* < 0.01 vs. miR-103-3p/miR-382-5p + ZIC4-NC group. **m**-**n** The co-effect of miR-103a-3p/miR-382-5p and ZIC4 on expression of Hsp90β by western blot assay. Data are presented as the means ± SD (*n* = 3, each group), **P < 0.01 vs. miR-103-3p / miR-382-5p + ZIC4 group; ^##^*P* < 0.01 vs. miR-103-3p/miR-382-5p + ZIC4-NC group

Firstly, we detected the mRNA and protein expressions of ZIC4 after overexpression or silencing of miR-103a-3p/miR-382-5p in GECs. As shown in Fig. 5a-d, the mRNA and protein expressions of ZIC4 were significantly decreased (0.4660 ± 0.1405/0.5360 ± 0.1016 folds and 0.3400 ± 0.0600/0.4098 ± 0.1060 folds) in the miR-103a-3p/miR-382-5p (+) group compared with those in the miR-103a-3p/miR-382-5p (+) NC group. However, the mRNA and protein expressions of ZIC4 were significantly up-regulated (1.830 ± 0.1751/1.6200 ± 0.1914 folds and 1.8700 ± 0.2422/2.1200 ± 0.3859 folds) in the miR-103a-3p/miR-382-5p (−) group compared with those in the miR-103a-3p/miR-382-5p (−) NC group. Subsequently, the potential binding sites of ZIC4 and miR-103a-3p/miR-382-5p were predicted with the help of bioinformatics databases. As expected, the relative luciferase activity in the ZIC4 Wt + miR-103a-3p/miR-382-5p (+) group was significantly impaired than that in the ZIC4 Wt + miR-103a-3p/miR-382-5p (+) NC group, while the relative luciferase activity in the ZIC4 Mut + miR-103a-3p/miR-382-5p (+) group was not affected (Fig. 5e, f). Then, GECs were co-transfected with miR-103a-3p/miR-382-5p agomir and ZIC4 (with or without 3’-UTR) plasmid and detected the angiogenesis. As shown in Fig. 5g–l, the cell viability, migration, and tube formation of GECs in miR-103a-3p/miR-382-5p + ZIC4 (non-3’UTR) group were markedly restored compared with that in miR-103a-3p/miR-382-5p + ZIC4 group (*P* < 0.01). Furthermore, we demonstrated that Hsp90β levels were prominently increased in miR-103a-3p/miR-382-5p + ZIC4 (non-3’UTR) group compared with miR-103a-3p/miR-382-5p + ZIC4 group (*P* < 0.01, Fig. 5m, n).

### ZIC4 overexpression prompted the angiogenesis of GECs by activating Hsp90β expression

**Fig. 6:**
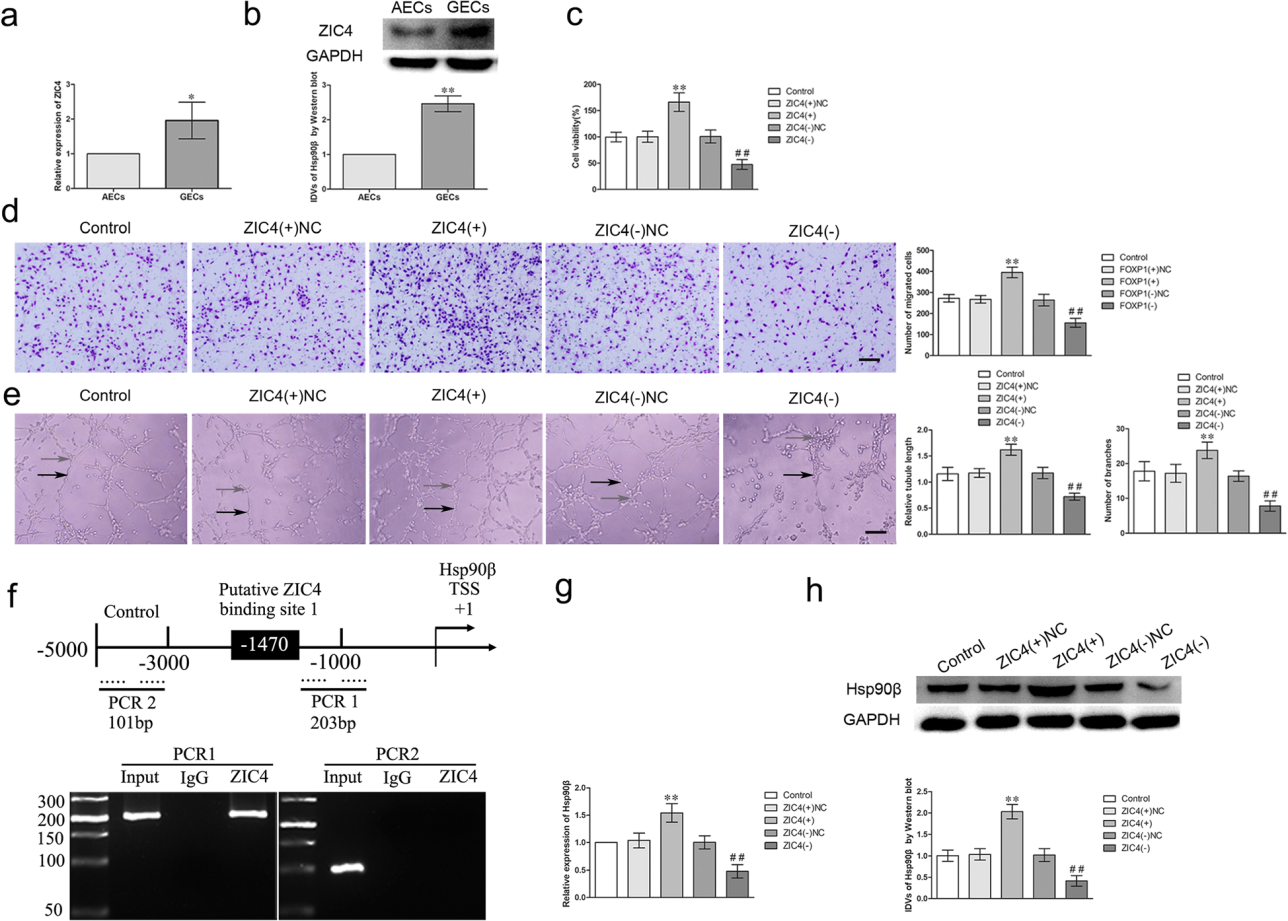
The expression of ZIC4 in GECs and ZIC4 regulated the viability, migration and tube formation of GECs by activating Hsp90β expression. **a**-**b** The mRNA and protein expressions of ZIC4 in AECs and GECs were evaluated by qRT-PCR and western blot. Data represent means ± SD (*n* = 5, each group). *P < 0.05, **P < 0.01 vs. AECs group. **c** ZIC4 regulated the viability of GECs. Values are means ± SD (*n* = 5, each group). **P < 0.01 vs. ZIC4 (+) NC group; ^##^*P* < 0.01 vs. ZIC4 (−) NC group. **d** ZIC4 regulated the migration of GECs. Values are means ± SD (*n* = 5, each group). **P < 0.01 vs. ZIC4 (+) NC group; ^##^*P* < 0.01 vs. ZIC4 (−) NC group. Scale bar represents 30 μm. **e** ZIC4 regulated the tube formation of GECs (black arrows, tube structures; gray arrows, tube branches). Values are means ± SD (*n* = 5, each group). **P < 0.01 vs. ZIC4 (+) NC group; ^##^*P* < 0.01 vs. ZIC4 (−) NC group. Scale bar represents 30 μm. **f** Schematic representation of human Hsp90β promoter region in 3000 bp upstream or 200 bp downstream of transcription start site (designated as + 1). ChIP PCR products for putative ZIC4 binding sites and an upstream region not expected to associate with ZIC4 are depicted with bold lines. **g**-**h** The mRNA and protein expressions of Hsp90β were detected by qRT-PCR and western blot after overexpression or silencing of ZIC4. Values are means ± SD (*n* = 5, each group). **P < 0.01 vs. ZIC4 (+) NC group; ^##^*P* < 0.01 vs. ZIC4 (−) NC group

The expression of ZIC4 in AECs and GECs was detected by qRT-PCR and western blot after confirming that ZIC4 is a downstream target of miR-103a-3p/miR-382-5p. As shown in Fig. 6a, b, the mRNA and protein expressions of ZIC4 were up-regulated (1.958 ± 0.5264-fold; 2.088 ± 0.2839-fold) in GECs compared with AECs. Consequently, overexpression or knockdown of ZIC4 was performed to investigate whether ZIC4 regulates angiogenesis of GECs and the transfection efficiency was validated by western blot (Additional file 2: Figure S2I). The viability, migration and tube formation of GECs were significantly increased in ZIC4 (+) group compared with ZIC4 (+) NC group; but the ZIC4 (−) group presented the opposite effects (Fig. 6,c–e *P* < 0.01). Furthermore, chromatin immunoprecipitation (ChIP) assays were performed to clarify whether ZIC4 directly bound to the promoters of Hsp90β in GECs. We further utilized JASPA database to propose there was a binding set between ZIC4 and Hsp90β protein, and we predicted the promoter sequence of Hsp90β and transcription start sites (TSSs) at the same time. Then we identified the potential binding site by scanning the DNA sequence from 3000 bp region upstream and 200 bp region downstream of TSS. Simultaneously, as shown in Fig. 6f, ZIC4 directly bound to the promoter region of Hsp90β in GECs, while in the corresponding negative control group, there was no combination between ZIC4 and the control region. The above results demonstrated there was a direct association between ZIC4 and the promoter sequence of Hsp90β in GECs. Subsequently, the mRNA and protein expressions of Hsp90β were detected in GECs after ZIC4 overexpression and silencing. The overexpression of ZIC4 up-regulated the expression of Hsp90β, whereas silencing of ZIC4 down-regulated its expression (Fig. 6g, h). These results indicated that ZIC4 promotes the angiogenesis of GECs by transcriptionally up-regulating Hsp90β.

### Hsp90β promoted the angiogenesis of GECs

**Fig. 7:**
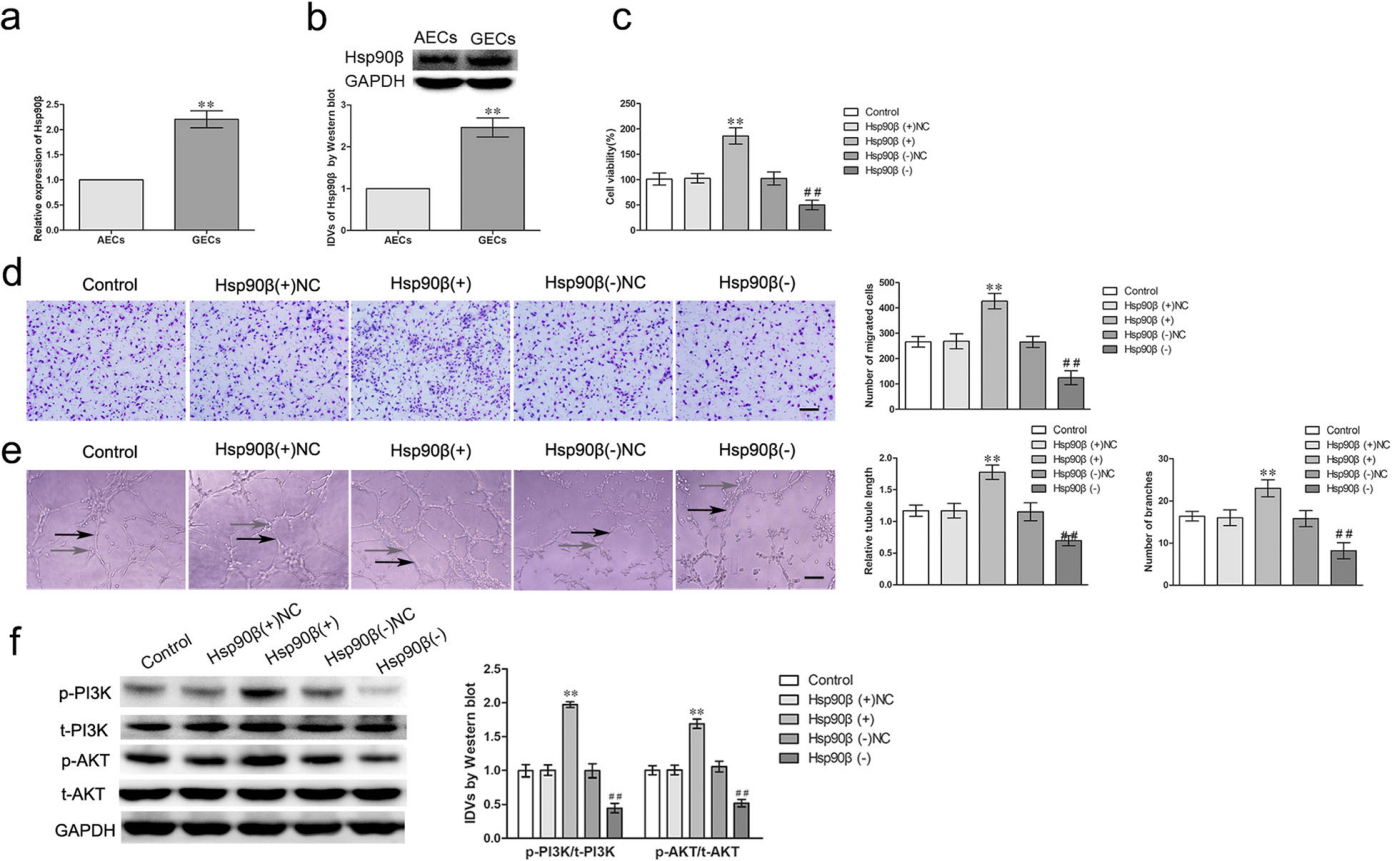
The expressions of Hsp90β in GECs and Hsp90β regulated the viability, migration and tube formation of GECs via PI3K/AKT pathway. **a**-**b** The mRNA and protein expressions of Hsp90β in AECs and GECs were evaluated by qRT-PCR and western blot. Data represent means ± SD (*n* = 5, each group). **P < 0.01 vs. AECs group. **c** The effect of Hsp90β knockdown on the viability of GECs were detected by CCK-8 assay. Values are means ± SD (*n* = 5, each group). **P < 0.01 vs. Hsp90β (+) NC group; ^##^*P* < 0.01 vs. Hsp90β (−) NC group. **d** The effect of Hsp90β knockdown on the migration of GECs were determined by Transwell assay. Values are means ± SD (*n* = 5, each group). **P < 0.01 vs. Hsp90β (+) NC group; ^##^*P* < 0.01 vs. Hsp90β (−) NC group. Scale bar represents 30 μm. **e** The effect of Hsp90β knockdown on the tube formation of GECs were evaluated by Matrigel tube formation assay. Values are means ± SD (*n* = 5, each group). **P < 0.01 vs. Hsp90β (+) NC group; ^##^*P* < 0.01 vs. Hsp90β (−) NC group. Scale bar represents 30 μm. **f** The protein expressions of PI3K/AKT in GECs were determined by western blot. Values represent the means ± SD (*n* = 5, each group). **P < 0.01 vs. Hsp90β (+) NC group; ^##^*P* < 0.01 vs. Hsp90β (−) NC group

The mRNA and protein expressions of Hsp90β were up-regulated (2.205 ± 0.3364 folds; 2.463 ± 0.2272 folds) in GECs compared with AECs (*P* < 0.01) by qRT-PCR and western blot (Fig. 7a, b). Consequently, the effects of Hsp90β overexpression or knockdown on GECs were investigated and the transfection efficiency was validated by western blot (Additional file 2: Figure S2J). As shown in Fig. 7c-e, overexpression of Hsp90β increased the cell viability, migration, and tube formation of GECs, knockdown of Hsp90β produced the opposite effects (*P* < 0.01). To investigate whether HSP90β promoted the angiogenesis of GECs by activating phosphatidylinositol 3-kinase (PI3K)/AKT pathways, the protein expressions of PI3K/AKT and ERK1/2 were evaluated in GECs. As shown in Fig. 7f, the expressions of p-PI3K/t-PI3K and p-AKT/t-AKT were significantly increased in the Hsp90β (+) group compared with the Hsp90β (+) NC group and knockdown of Hsp90β produced the opposite effects (*P* < 0.01).

### MOV10 knockdown combined with circ-DICER1 knockdown suppressed glioma angiogenesis in vivo

**Fig. 8:**
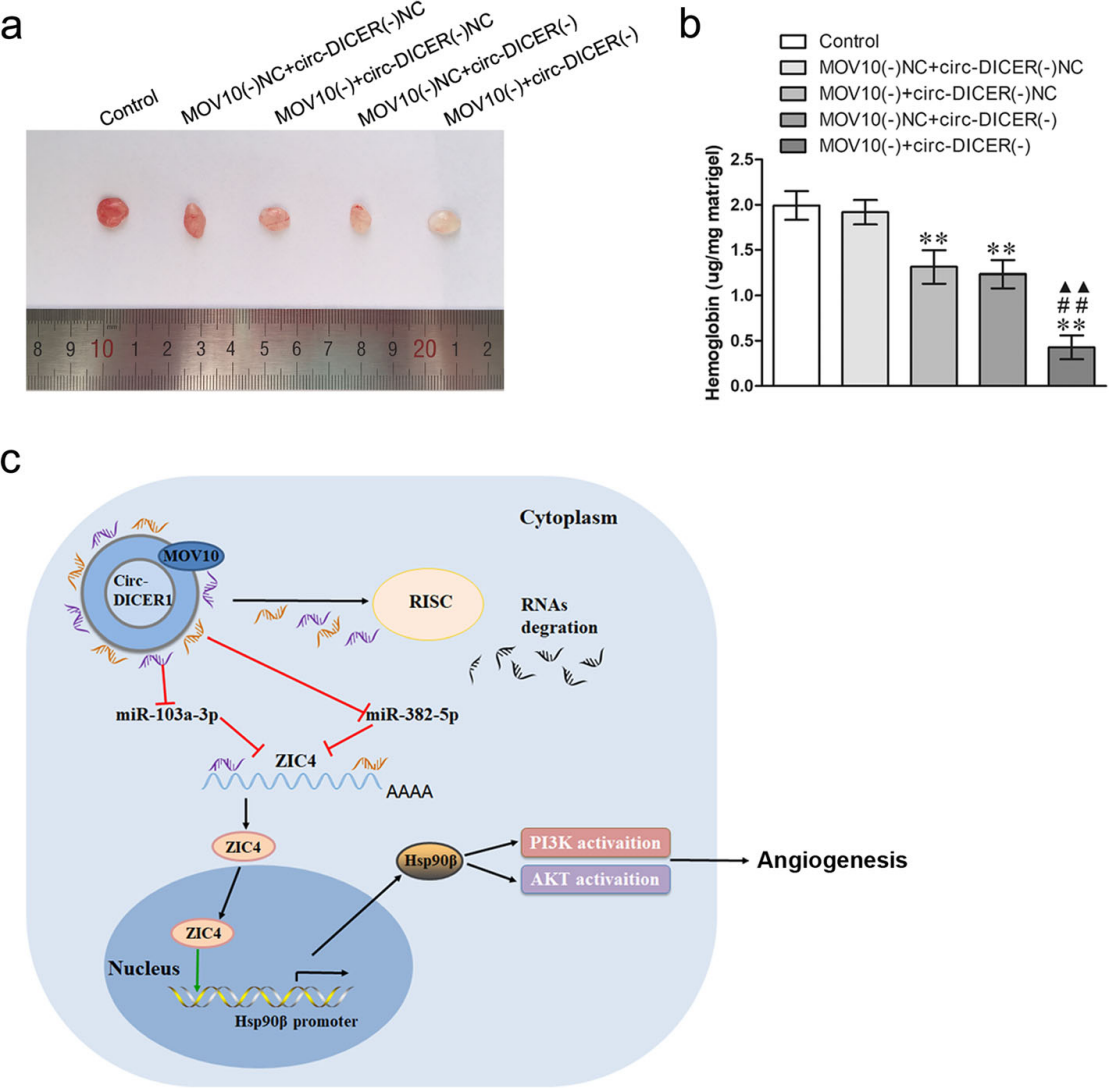
Knockdown of both MOV10 and circ-DICER1 suppressed the angiogenesis in vivo. **a** The co-effect of MOV10 and circ-DICER1 on the angiogenesis in vivo was evaluated by Matrigel plug assay. **b** The amount of hemoglobin was measured after combination of MOV10 and circ-DICER1. Data represent the means ± SD (*n* = 5, each group). **P < 0.01 vs. MOV10 (−) NC + circ-DICER1 (−) NC group; ^##^*P* < 0.01 vs. MOV10 (−) + circ-DICER1 (−) group; ^▲▲^*P* < 0.01 vs. MOV10 (−) NC + circ-DICER1 (−) group

To assess whether MOV10 and circ-DICER1 are able to regulate GECs angiogenesis in vivo, matrigel plug assay was performed. As shown in Fig. 8a, b, compared with the MOV10 (−) NC + circ-DICER1 (−) NC, the hemoglobin content in Matrigel plug was lower in MOV10 (−) + circ-DICER1 (−) NC group, MOV10 (−) NC + circ-DICER1 (−) group and MOV10 (−) + circ-DICER1 (−) group. Moreover, the MOV10 (−) + circ-DICER1 (−) group produced the lowest hemoglobin content. Finally, the schematic cartoon underlying the mechanism of circ-DICER1 on the angiogenesis of GECs was presented in Fig. 8c.

## Discussion

The role of circRNAs in the development and progression of tumor has drawn growing attention from researchers. Studies have shown that RNA binding proteins can interact with circRNAs. Simon et al. found that RNA binding protein Quaking promoted the synthesis of multiple circRNAs during the epithelial mesenchymal transformation of human breast epithelial cells [30]. And circ-Foxo3 retards cell cycle progression via forming ternary complexes with p21 and CDK2 [31]. Recent report has clarified that MOV10 provided antiviral activity against RNA viruses by enhancing interferon induction [32], and MOV10 silencing can increase the expression of tumor suppressor INK4a [33]. In melanoma, inhibition of MOV10 increases the synthesis and secretion of Wnt 5a to further affect the metastasis of melanoma cells [34]. However, the regulation of MOV10 in GECs has not yet been reported. This present study investigated the expression of MOV10 was up-regulated in GECs, and knockdown of MOV10 inhibited the cell activity, migration, and tubular formation of GECs. In addition, the binding site of MOV10 and circ-DICER1 was confirmed with the help of CircInteracome database, and the result of RIP demonstrated MOV10 promoted the angiogenensis of glioma by targeting circ-DICER1.

Studies have reported the expressions of circRNAs were abnormal in a variety of tumor tissues. In esophageal squamous cell carcinoma tissues and cells, the expression of hsa_circ_0067934 is up-regulated and promoted proliferation [35]. And has_circ_002059 was found to be significantly down-regulated in gastric cancer tissues compared with paired adjacent nontumorous tissues, and was closely related to multiple clinical pathological factors in gastric cancer patients [36]. The above findings indicate abnormal expressed circRNAs play an important role in the development and progression of tumor. This study found that the expression of circ-DICER1 was up-regulated in GECs, and knockdown of circ-DICER1 inhibited the cell activity, migration, and tubular formation of GECs, which suggested circ-DICER1 plays a carcinogenic role in GECs. Other circRNAs can play the same role in glioma cells or GECs. For example, circ-SHKBP1 was highly expressed in GECs, and silencing of circ-SHKBP1 inhibited the cell viability, migration and tube formation of GECs [37]. And silencing of circ-cZNF292 suppresses tube formation of human glioma cells via the Wnt/beta-catenin signaling pathway [38]. Studies have shown that the expression of Dicer was different in many tumors and is related to the prognosis of patients [14, 15]. This study proved that there was no statistical difference in the expression of Dicer 1 in normal cerebral microvascular endothelial cells and GECs. RNase R degraded linear Dicer 1, but did not affect the expression of circ-DICER1. Therefore, it has been assumed that circ-DICER1 and linear Dicer1 act alone and do not affect each other, which is consistent with the feature of circ-SHKBP1 [37].

It has been found that a variety of circRNAs can act as competing endogenous RNAs (ceRNAs) to bind to miRNAs, and act as miRNAs sponges [11]. CirkIPK3 sponged multiple miRNAs and regulated the proliferation of liver cancer cells by direct binding miR-124 [39]. And circ-TTBK2 regulates the proliferation, migration, and invasion of glioma cells by targeting miR-217 [40]. In addition, circ-TCF25 regulates the proliferation, migration, and invasion of bladder cancer cells via miR-103a-3p/miR-107-CDK6 pathway [41]. And circ-SHKBP1 act as a “molecular sponge” to bind with miR-544a and miR-379 respectively and regulate the functions of GECs [37]. It has been proved the binding sites between circ-DICER1 and miR-103a-3p/miR-382-5p with the help of Starbase. And our data further confirmed the above binding sites. In addition, the RIP assay showed that circ-DICER1 and miR-103a-3p or miR-382-5p was respectively present in the RNA induced silence complex (RISC). Further studies have shown that silencing of circ-DICER1 and overexpression of miR-103a-3p/miR-382-5p significantly reduced the cell viability, migration, and tube formation of GECs; meanwhile, silencing of both circ-DICER1 and miR-103a-3p/miR-382-5p reversed the decline in the cell viability, migration and tube formation of GECs caused by circ-DICER1 silencing, which indicates that the roles of circ-DICER1 in GECs are regulated by combining miR-103a-3p/miR-382-5p. It is suggests that circ-DICER1 sponges miR-103a-3p/miR-382-5p to further regulated the functions of GECs.

This study further proved that miR-103a-3p/miR-382-5p were lowly expressed and played a suppressor role in GECs. Studies have confirmed that miR-103a-3p is an important molecule in the process of glioblastoma [19]. And miR-103a-3p plays a tumor suppressor role in a variety of tumors, such as glioma [18] and bladder cancer [41]. Similar to our findings, miR-103a-3p promoted the apoptosis of human colorectal adenocarcinoma associated endothelial cells and inhibited the angiogenesis of tumors [20]. In addition, miR-382-5p plays an inhibitory role in most tumors, such as non-small cell lung cancer and colon cancer [21, 22]. Studies have shown that miR-382-5p is low expression in glioma cells, and SETD8 promotes cell proliferation and invasion by targeting miR-382-5p [42]. In addition, high expression of miR-382 induced by hypoxia promotes the proliferation, migration, and tube formation of endothelial cells in gastric cancer [23]. According to the above findings, miR-103a-3p and miR-382-5p play the role of tumor suppressor in glioblastoma tissues or cells as well as in GECs. MiRNAs target the 3’UTR of mRNA to inhibit mRNA translation by completely or not fully complementary pairing [16]. Researches have confirmed that AGO1 [43], FEZF1 [18] and CDK6 [41] are target genes of miR-103a-3p; and GOLM1 [44], EZH2 [45] and HSPD1 [46] are target genes of miR-382-5p. Our findings proved ZIC4 is a target gene of miR-103a-3p /miR-382-5p, and miR-103a-3p/miR-382-5p regulated the angiogenesis of glioma by targeting ZIC4.

The transcription factor ZIC4 is one member of C2H2 zinc finger protein ZIC4 family, and it plays an important role in neurodevelopment. It has been reported that ZIC4 is highly expressed in myeloblastoma tissues [47]. Meanwhile, the expression of ZIC4 is up-regulated in glioma tissues by detecting TCGA database. In this study, the expression of ZIC4 in GECs increased significantly compared to that of AECs. Additionally, ZIC4 overexpression promotes the cell viability, migration, and tube formation of GECs, which indicates ZIC4 may play a carcinogenic role in GECs. Furthermore, there are the binding sites of ZIC4 in the promoter region of Hsp90β after the prediction analysis of JASPAR CORE database. As expected, the results of CHIP confirmed that ZIC4 can be combined with the promoter region of Hsp90β to regulate its transcription.

Hsp90β is a member of the heat shock protein family. Most of its customer proteins are closely related to cell proliferation, invasion, metastasis and angiogenesis, such as CDK4, AKT and so on [48, 49]. A number of studies shown that Hsp90 inhibitors decreased the expression of VEGFR-1, inhibited the migration and tube formation of endothelial cell to further inhibit tumor angiogenesis in various cancers such as colorectal adenocarcinoma [50]. Moreover, Hsp90β inhibitors can inhibit the migration and invasion of glioma cells and inhibit the secretion of vascular endothelial growth factor [28]. In addition, other studies clarified that Hsp90 β inhibits the apoptosis of intestinal cells by activating Akt pathway [51]. The above findings are consistent with our findings. The expression of Hsp90β was increased in GECs and promoted the cell viability, migration, and tube formation of GECs by activating the PI3K/Akt pathway. The above evidence indicates that miR-103a-3p/miR-382-5p affected the regulation of ZIC4 on its target gene Hsp90β to further affect the angiogenesis of glioma by targeting and negatively regulating the expression of ZIC4.

## Conclusions

The present study demonstrated that the expressions of RNA binding proteins MOV10, circ-DICER1, ZIC4, and Hsp90 β were up-regulated in GECs, while miR103a-3p/ miR-382-5p were down-regulated in GECs for the first time. In GECs, MOV10 combines to circ-DICER1, and circ-DICER1 acts as a molecular sponge to adsorb miR-103a-3p/miR-382-5p and weakens the negative regulation of miR-103a-3p/miR-382-5p on ZIC4 to further up-regulate the expression of ZIC4. ZIC4 increases the expression of its downstream target gene Hsp90 β and Hsp90 promotes the cell viability, migration, and tube formation of GECs by activating PI3K/Akt signaling pathway. Consequently, MOV10/circ-DICER1/miR -103a-3p (miR-382-5p)/ZIC4 pathway plays a vital role in regulating the angiogenesis of glioma. Comparatively, the study in vivo and in vitro demonstrated that the effects of both MOV10 and circ-DICER1 silencing on the angiogenesis of glioma were better than the effects of MOV10 or circ-DICER1 alone silencing. Our findings provides not only novel mechanisms for the study of glioma angiogenesis, but also new potential targets for anti-angiogenesis therapies of glioma in the view of multiple molecular control network of MOV10 (RNA binding protein), circ-DICER1 (circRNAs), miR-103a-3p/miR-382-5p (miRNAs), ZIC4 (transcription factor), and Hsp90 β (target gene).

## Additional files

Additional file 1: **Figure S1.** RNA binding protein microarrays data, transfection efficiency of shMOV10 and circRNA microarrays data. (A) RNA binding protein gene expression profiles as obtained from samples in three groups as indicated. (B) The transfection efficiency of shMOV10 was detected by western blot. Data represent means ± SD (*n* = 5, each group). **P < 0.01 versus. MOV10 (−) NC group. (C) circRNA gene expression profiles as obtained from samples in three groups as indicated. Note: The circRNA ID is in Pubmed. (TIF 721 kb)

Additional file 2: **Figure S2.** The transfection efficiency of shcirc-DICER1, miR-103-3p/miR-382-5p, ZIC4 and Hsp90β, and miRNA microarrays data. (A) The transfection efficiency of circ-DICER1 knockdown was detected by qRT-PCR. Data represent means ± SD (*n* = 5, each group). **P < 0.01 versus. Circ-DICER1 (−) NC group. (B) The expression of DICER1 was measured after knockdown of circ-DICER1. Data represent means ± SD (*n* = 5, each group). (C) The mRNA expression of DICER1 was detected by qRT-PCR after DICER1 knockdown. Data represent means ± SD (*n* = 5, each group). **P < 0.01 versus. sh-DICER1. (D) The expression of circ-DICER1 was measured after knockdown of DICER1. Data represent means ± SD (*n* = 5, each group). (E) MiRNA gene expression profiles as obtained from samples in three groups as indicated. (F) The binding sites between DICER1 and miR-103a-3p were predicted, and the relative luciferase activity was evaluated in HEK293T cells. Data represent means ± SD (*n* = 5, each group). **P < 0.01 versus DICER1 Wt + miR-103a-3p (+) NC group. (G-H) The transfection efficiency of miR-103a-3p (G) and miR-382-5p (H) agomir or antagomir were evaluated by qRT-PCR. Data represent means ± SD (*n* = 5, each group). **P < 0.01 versus. miR-103a-3p / miR-382-5p (+) NC group, ##*P* < 0.01 versus. miR-103a-3p / miR-382-5p (−) NC group. (I) The transfection efficiencies of ZIC4 were evaluated with western blot. Data represent means ± SD (*n* = 5, each group). **P < 0.01 versus. ZIC4 (+) NC group, ##*P* < 0.01 versus. ZIC4 (−) NC group. (J) The transfection efficiency of Hsp90β was investigated with western blot. Data represent means ± SD (*n* = 5, each group). **P < 0.01 versus. Hsp90β (+) NC group, ##*P* < 0.01 versus. Hsp90β (−) NC group. (TIF 780 kb)

## Abbreviations

3’UTR: 3′untranslated region
CCK-8: Cell counting kit-8
ChIP: Chromatin immunoprecipitation
circ-DICER1: Circular DICER1
circRNAs: Circular RNAs
DMEM: Dulbecco’s modified Eagle’s medium
ECs: Endothelial cells
GECs: Glioma-exposed endothelial cells
hCMEC/D3: Human cerebral microvascular endothelial cell
HEK293T: Human embryonic kidney 293 T
Hsp90β: Heat shock protein 90β
IDVs: Integrated density values
miRNAs: MicroRNAs
MOV10: Moloney leukemia virus 10
NC: Negative control
RBPs: RNA binding proteins
RIP: RNA-Binding protein immunoprecipitation
RISC: RNA-induced silencing complex
siRNAs: Small interference RNAs
ZIC4: Zinc finger of the cerebellum 4
