# Supplementary figures and images for: MOV10 binding circ-DICER1 regulates the angiogenesis of glioma via miR-103a-3p/miR-382-5p mediated ZIC4 expression change
Source: J Exp Clin Cancer Res. 2019 Jan 8;38:9. doi: 10.1186/s13046-018-0990-1 (PMC6323715; doi:10.1186/s13046-018-0990-1)

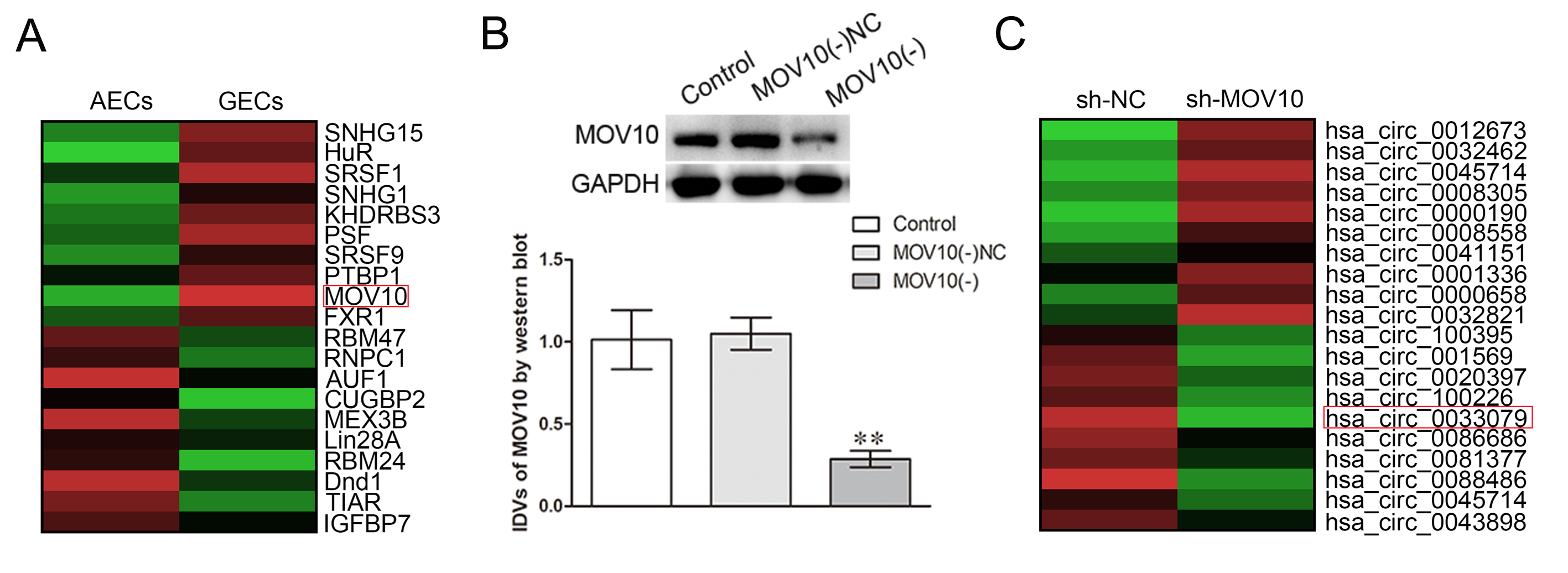

Supplement: Supplementary file 1 — Figure S1. RNA binding protein microarrays data, transfection efficiency of shMOV10 and circRNA microarrays data. (A) RNA binding protein gene expression profiles as obtained from samples in three groups as indicated. (B) The transfection efficiency of shMOV10 was detected by western blot. Data represent means ± SD (n = 5, each group). **P < 0.01 versus. MOV10 (−) NC group. (C) circRNA gene expression profiles as obtained from samples in three groups as indicated. Note: The circRNA ID is in Pubmed. (TIF 721 kb) [file 13046_2018_990_MOESM1_ESM.tif]

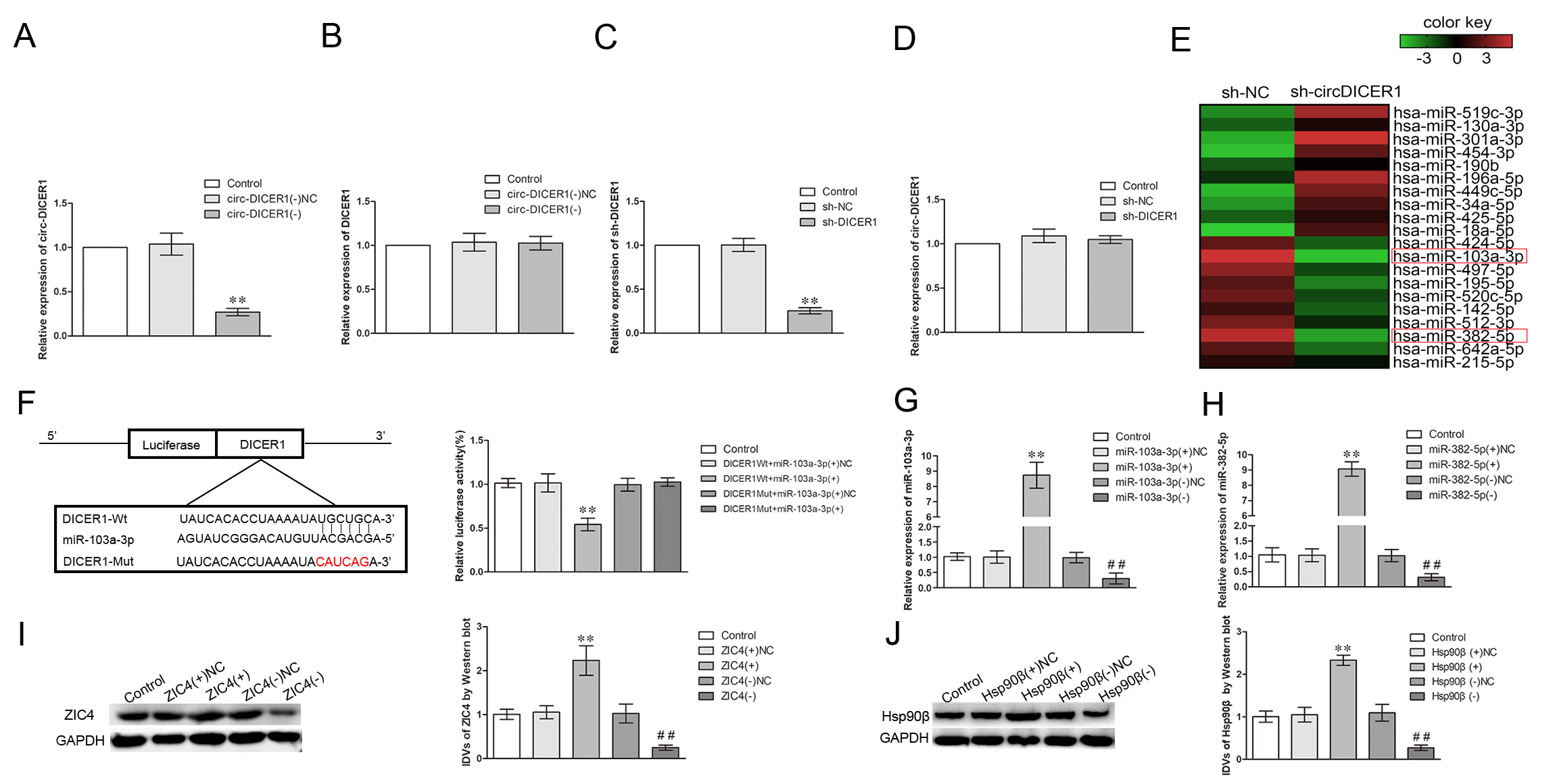

Supplement: Supplementary file 2 — Figure S2. The transfection efficiency of shcirc-DICER1, miR-103-3p/miR-382-5p, ZIC4 and Hsp90β, and miRNA microarrays data. (A) The transfection efficiency of circ-DICER1 knockdown was detected by qRT-PCR. Data represent means ± SD (n = 5, each group). **P < 0.01 versus. Circ-DICER1 (−) NC group. (B) The expression of DICER1 was measured after knockdown of circ-DICER1. Data represent means ± SD (n = 5, each group). (C) The mRNA expression of DICER1 was detected by qRT-PCR after DICER1 knockdown. Data represent means ± SD (n = 5, each group). **P < 0.01 versus. sh-DICER1. (D) The expression of circ-DICER1 was measured after knockdown of DICER1. Data represent means ± SD (n = 5, each group). (E) MiRNA gene expression profiles as obtained from samples in three groups as indicated. (F) The binding sites between DICER1 and miR-103a-3p were predicted, and the relative luciferase activity was evaluated in HEK293T cells. Data represent means ± SD (n = 5, each group). **P < 0.01 versus DICER1 Wt + miR-103a-3p (+) NC group. (G-H) The transfection efficiency of miR-103a-3p (G) and miR-382-5p (H) agomir or antagomir were evaluated by qRT-PCR. Data represent means ± SD (n = 5, each group). **P < 0.01 versus. miR-103a-3p / miR-382-5p (+) NC group, ##P < 0.01 versus. miR-103a-3p / miR-382-5p (−) NC group. (I) The transfection efficiencies of ZIC4 were evaluated with western blot. Data represent means ± SD (n = 5, each group). **P < 0.01 versus. ZIC4 (+) NC group, ##P < 0.01 versus. ZIC4 (−) NC group. (J) The transfection efficiency of Hsp90β was investigated with western blot. Data represent means ± SD (n = 5, each group). **P < 0.01 versus. Hsp90β (+) NC group, ##P < 0.01 versus. Hsp90β (−) NC group. (TIF 780 kb) [file 13046_2018_990_MOESM2_ESM.tif]
